# Which quality of life score is best for glaucoma patients and why?

**DOI:** 10.1186/1471-2415-8-2

**Published:** 2008-01-23

**Authors:** Philip Severn, Scott Fraser, Tracy Finch, Carl May

**Affiliations:** 1 Sunderland Eye Infirmary, Queen Alexandra Road, Sunderland, SR2 9HP, UK; 2 Institute of Health and Society, 21 Claremont Place, Newcastle University, Newcastle upon Tyne, NE2 4AA, UK

## Abstract

**Background:**

The glaucomas are generally asymptomatic diseases until they are very advanced. They affect 2% of the population over 40 years of age and therefore represent a significant public health issue. There have been a number of attempts to develop quality of life scales for the disease. This review discusses the pros and cons of these scales and suggests the best of the current ones for use in a clinical setting.

**Methods:**

Medline, Embase and Google Scholar were searched for relevant articles. No time period was defined and all types of article were included.

**Results:**

11 Quality of Life scores were identified that have been used with glaucoma patients.

**Conclusion:**

There is no generally accepted 'best' Quality of Life instrument for use in glaucoma. Many of the scales are biased towards physical symptoms and do little to address the personal or social factors of the disease. Further work is needed to produce scales that address all these areas as well as being simple to administer in a clinical setting.

## Which quality of life score is best for glaucoma patients and why?

Glaucoma is the term given to the chronic, debilitating, progressive group of eye disorders that can lead to visual field loss and blindness. Glaucoma usually produces certain characteristic visual field defects in the individual's peripheral, as well as central vision. Due to the intractable nature of the disease the patient usually spends, following diagnosis, the rest of their life attending an eye hospital and taking frequent (daily) ocular anti-hypertensive medication. The treatment has associated side effects, it is expensive and often inconvenient to instill. It has been reported that approximately 67 million patients suffer from glaucoma and roughly 10% of these are blind. It is therefore not surprising that glaucoma frequently has a large impact on a patient's quality of life [1].

The diagnosis of glaucoma affects people in different ways. Some readily accept the diagnosis and are keen to seek out information [2]. Others are more ignorant and disappear into the community, only to return years later with a marked deterioration in their visual function. Most patients fall in between the two extremes and adhere to their treatment in the main with little or no understanding of the disease process.

Physicians have long strived to quantify quality of life (QoL) in patients with glaucoma. The reasons for this range from understanding of the patients experience of the disease to the measurement of outcomes in treatment trials. However although a number of instruments have been used/developed it has proved difficult to develop the idea glaucoma QoL score. Patients can lose quality of life for a number of reasons. The distress of the diagnosis, the insidious loss of vision and independence, the problems with frequent treatment and regular hospital outpatient appointment reviews. So how do we assess the impact of the disease on the patient's quality of life? Does the QoL of life score need to be all encompassing? Should it include all aspects of the disease process from diagnosis to death? Unfortunately the QoL scales can be complicated, non user-friendly and contain a myriad of complex mathematics. The aim of this article is to give a narrative review of the readily available QoL scales, highlight the strengths and weaknesses of each and suggest which one we find "best" for our assessing glaucoma patients in clinical practice.

## Review strategy

We searched Medline, Embase and Google Scholar for relevant articles. We did not define any time period or any type of research (e.g. original, reviews, correspondence). Keywords used to search were:

Quality of Life Scores, Quality of life scales, QoL, eye disease, glaucoma.

Articles identified were accessed and references checked for any other potentially relevant literature.

Eleven pertinent articles pertaining to readily available glaucoma QoL instruments were identified. The Medical Outcomes Study (Mos-20) has been incorporated into the MOS SF-36 questionnaire and therefore excluded from discussion.

## Selecting a QoL scale

When selecting a QoL scale for a glaucoma patient we might hope the instrument fulfils the following criteria:

1. Ease of use in a clinical setting

2. Contains minimal complex mathematics

3. Allows reproducible data to be obtained

4. Correct underlying principles pertaining to glaucoma

5. Simple understandable questions with unambiguous answers

There are a number of well-documented tools that have been used to quantify the subjective status of glaucoma patients [3,4]. These include:

• Generic instruments i.e. not disease state specific. (SF-36 [5], SIP [6])

• Vision specific instruments (VF-14 [7], NEI-VFQ [8], NEI-VFQ-25 [9], ADVS [10])

• Glaucoma-specific instrument (GSS [11], COMTOL [12], GQL-15 [13], SIG [14])

It is perhaps not surprising that the glaucoma specific instruments act as a greater discriminator between glaucoma patients and controls. There appears to be a stronger relationship with objective (clinical) measures of disease state than the generic instruments. Many of the glaucoma instruments assess symptoms of glaucoma and effects on activities, but do not include assessment of the *importance *of such impacts for the individual. QoL assessment in glaucoma has been developed further in a handful of studies that have undertaken utility-based approaches, such as time trade-off (how much life expectancy would patients be prepared to give up in exchange for absence of symptoms), and conjoint analysis (indicating preferences amongst pairs of options to arrive at rankings of importance of health/disease states). Such approaches generally indicate ratings of subjective health status that are more negative than non-glaucoma patients (but not greatly so), but which are much more positive than patients who are blind. This appears to reflect the nature of disease progression in glaucoma, as many patients have little or no symptoms in the early stages of disease.

In order to assess the strengths and weaknesses we will look at these in more detail.

## The Medical Outcomes Study Short Form-36 (SF-36) [5]

The SF-36 (The Medical Outcomes Study Short Form-36) is a multi-purpose medical health survey containing 36 questions. The questionnaire is easy to use, takes roughly 10 minutes to complete, and is considered reliable. Unfortunately there is a weak correlation between all SF-36 domains and visual acuity or visual field impairment, which is the end-point of any measure of glaucoma therapy. Therefore, it is not felt that the SF-36 is a suitably robust assessment tool in glaucoma patients [4].

## The Sickness Impact Profile (SIP) [6]

The main aim of the SIP (The Sickness Impact Profile) development was to provide a measurable instrument of perceived health status that is robust enough to detect changes or differences in health status that occur in time or between groups [6]. There are 136 categories, 12 domains and takes in excess of 30 minutes to complete. Although the validity and reliability of SIP has been researched it suggested that the instrument is not easy to use, takes too long to perform and is therefore considered unpractical for use in the clinical setting.

## The VF-14 [7]

The VF-14 aims to measure functional impairment in patients with cataracts. There are 14 functional activity questions and the instrument is technically straightforward to use. It has high internal consistency and has a stronger self-rating correlation than the SIP score. It has moderate relevance to glaucoma patients in terms of assessment of visual acuity. However, due to the exclusion of visual field defect and colour vision, two key indicators of optic nerve assessment and therefore effectiveness of glaucoma therapy, the instrument has largely been disregarded.

## The National Eye Institute Visual Function Questionnaire (NEI-VFQ) [8]

The NEI-VFQ (The National Eye Institute Visual Function Questionnaire) is a 51 item, 12-domain tool that is not particularly easy to use. The instrument takes 15 minutes to use. It has been reported that it is more sensitive than the SF-36 in differentiating patients with glaucoma [4]. The NEI-VFQ is fully validated and is a widely used tool that allows vision-dependent tasks to be assessed.

## The 25-item National Eye Institute Visual Function Questionnaire (NEI-VFQ-25) [9]

The NEI-VFQ-25 (The 25-item National Eye Institute Visual Function

Questionnaire) is a 25 item, 12-domain tool that appears to be an improved version of its predecessor in that it has a more clinical emphasis. The instrument takes roughly 5 minutes to use, is reliable and fully validated. However, the lack of visual field consideration causes this tool to fall down in comparison to some of the more specific glaucoma tools. The NEI-VFQ-25 is easy to use which explains why it has been translated into many languages and forms the basis of a number of ocular studies. The NEI-VFQ and NEI-VFQ-25 are used as benchmarks against which more specific glaucoma QoL tools are compared.

## The Activities of Daily Vision Scale (ADVS) [10]

The ADVS (The Activities of Daily Vision Scale) is a 20 item, 12-domain tool that is easy to use. It is reliable and has good internal consistency [3]. However, it is a cataract driven tool and does not take into account the loss of peripheral vision. Although there is good correlation between other factors, such as visual acuity and visual field, the exclusion of peripheral field makes it less pertinent to glaucoma patients.

## The Glaucoma Symptom Scale (GSS) [11]

The GSS (The Glaucoma Symptom Scale) is a 10 item, 2-domain tool. It uses a cross-section of symptoms, functional impairment, and vision-targeted health related quality of life assessments among patients with glaucoma [3]. It is reliable, has good internal consistency, and is short and easy to use. The GSS was able to discriminate between persons with and without glaucoma. However, the instrument did not demonstrate association with Esterman (binocular) visual field changes. Also, the tool did not address treatment related factors relevant to QoL in glaucoma patients.

## The Comparison of Ophthalmic Medication for Tolerability (COMTOL) [12]

The COMTOL (The Comparison of Ophthalmic Medication for Tolerability) is a 37 item, 13-domain tool with 4 global questions. This tool is specific for ophthalmic medication tolerability and has good internal consistency, reproducibility and reliability. The role as a general tool is therefore very limited.

## The Glaucoma Quality of Life-15 (GQL-15) [13]

The GQL-15 (The Glaucoma Quality of Life-15) is a 15 item, 4-domain tool that is short and easy to use. The instrument is based on the premise that perceived visual disability (dark adaptation, disability glare, outdoor mobility tasks and activities using peripheral vision) is significantly associated with binocular visual field loss.

It has good internal consistency and reliability. The tool has been shown to demonstrate that difficulties in everyday life are mirrored by poor performance in a number of psychophysical tests [3]. The tool does concentrate on the physical impact of the disease process and does not address the broader QoL factors. However, if these factors are addressed then the instrument becomes less user-friendly in clinical practice.

## The Symptom Impact Glaucoma Score (SIG) and Glaucoma Health Perceptions index (GHPI) [14]

The SIG (The Symptom Impact Glaucoma Score) is a 43 item, 4-domain tool. The GHPI (Glaucoma Health Perceptions index) contains four items that address the physical, emotional, social and stresses associated with living with glaucoma. The SIG and GHPI were designed for the CIGTS study. These instruments are excellent research tools. Both display demonstrable validity and reliability. However, they were primarily designed for research and appear to have limited clinical crossover relevance. The interviewers underwent an intensive 10-hour training course before patient interaction commenced.

## Conclusion

There are multiple tools/instruments that have been used to assess the QoL in glaucoma patients. Despite many reviews there does not appear to be an accepted "industry standard" tool. The NEI-VFQ and NEI-VFQ-25 remain the benchmark against which new glaucoma QoL are compared. Many of the tools have their place but often rely heavily on the physical symptoms rather than considering social and personal factors.

No one QoL scale has been shown to be ideal and more research is required to define a more precise and user-friendly instrument for use in glaucoma patients. Glaucoma is a disease that is asymptomatic until its very late stages thus sufferers can be transformed from considering themselves 'normal' to becoming patients even in the absence of any obvious functional impairment. This does of course make assessment of QoL much harder and scales need to account of this. Personal factors such as worry, self-identity (and the change the disease state makes to this), inconvenience of treatment, financial impact of treatment or alteration in employment/driving need to be addressed.

We are aware that the generic problem with many of the QoL instruments is their parochial bias. This is currently being addressed and will require further review in future years in order to assess the impact of these non-Western based QoL instruments [4].

One of the aims of this review was to suggest the most useful QoL scale currently available. One of the key aims when considering the usability of a QoL tool is to assess its ease of use. When the QoL instrument attempts to glean too much information they have a tendency to become less user-friendly. Ultimately, we feel the NEI-VFQ and NEI-VFQ-25 will and should remain the comparative benchmark for QoL tools. The GQL-15 is probably the most useful and clinically relevant tool and the SIG is the most appropriate research tool.

## Competing interests

The author(s) declare that they have no competing interests.

## Authors' contributions

All four authors contributed to the conception of the study and were involved in writing the manuscript. PS searched and retrieved the literature.

## Pre-publication history

The pre-publication history for this paper can be accessed here:


